# Positronium Atoms Solvated in Liquid Alcohols: A Multicomponent Quantum Mechanics/Molecular Mechanics Approach

**DOI:** 10.1021/acs.jpcb.5c06150

**Published:** 2025-11-20

**Authors:** Leonardo Martins, Mateus Bergami, Jorge Charry, Andres Reyes, Kaline Coutinho, Márcio T. Do N. Varella

**Affiliations:** † Instituto de Física, 61755 Universidade de São Paulo, Rua do Matão 1371, CEP, São Paulo, São Paulo 05508-090, Brazil; ‡ Department of Engineering and Physics, 4209 Karlstad University, Karlstad SE-65188, Sweden; § Department of Physics and Materials Science, University of Luxembourg, Luxembourg City, L-1511, Luxembourg; ∥ Department of Chemistry, 28021 Universidad Nacional de Colombia, Av. Cra. 30 #45-03, Bogotá 111321, Colombia

## Abstract

Positronium (Ps) atoms are highly sensitive probes of condensed-phase structure and dynamics, yet their theoretical description in complex molecular environments remains challenging. We present an extension of our QM/MM protocol to investigate Ps in methanol and ethanol, building on our earlier study in water. Classical Monte Carlo simulations employing newly parametrized Ps-solvent force fields reveal well-defined cavity structures, whose sizes are consistent with hydrated Ps and systematically smaller than those predicted by empirical bubble models. Multicomponent quantum calculations employing the Any Particle Molecular Orbital (APMO) method have identified physically meaningful cavity states, characterized by substantial electron-positron overlap, and have ruled out spurious surface states as artifacts of the QM region truncation. While vertical detachment energies are insensitive primarily to solvent structure, computed pick-off annihilation lifetimes showed a marked dependence on cavity size. Employing orbital-dependent enhancement factors, our results achieve good agreement with experimental PALS data for both solvents. This study demonstrates the transferability and predictive power of our QM/MM protocol for Ps, providing a framework that can be systematically extended to more complex solvents and biological environments, thereby advancing the theoretical interpretation of Ps annihilation in complex systems.

## Graphical abstract

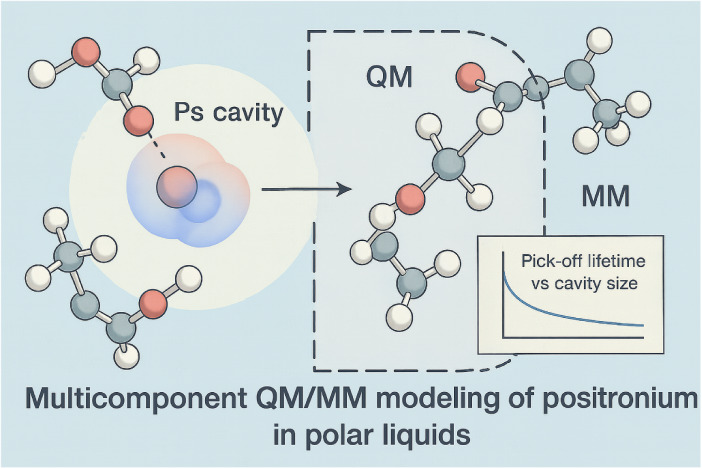

## 1 Introduction

Positrons and positronium (Ps) atoms are exceptional condensed matter probes that have led to several advances in materials science and medical applications.^1,2^ The sensitivity of pair annihilation to the presence of voids and to the chemical environment allows for the characterization of metals,^3^ semiconductors,^4–6^ ionic liquids,^7–9^ polymers,^10,11^ soft matter,^12–14^ and living biological systems.^15^ Positron Annihilation Spectroscopy (PAS) techniques^1,13,16^ are often used for characterization, while Positron Emission Tomography (PET) is used for medical imaging, cancer diagnosis, and tumor detection.^17–19^

High-energy positrons (∼ 10^2^ keV) are typically produced by β^+^ decay. Once injected in condensed systems, they thermalize through a complex cascade of inelastic interactions.^20,21^ In liquids, a fast positron induces ionization events producing energetic electrons, which, in turn, further ionize the medium, generating “spurs”, i.e., nano volumes containing electron–ion pairs. As the positrons slow down, the spur domains get closer together, eventually producing “blobs”, i.e., regions where the positron motion becomes diffusive. Electron–ion pairs are also present in the blob, where Ps formation usually takes place. The quasi-free Ps atoms are energetically stable against the breakup into hydrated electrons and positrons, and eventually diffuse back into the liquid.^22^ The Ps atoms may be formed in a singlet state, called para-Ps (p-Ps), or a triplet state, called ortho-Ps (o-Ps). The gas-phase lifetimes are 125 ps and 142 ns, respectively, for p-Ps and o-Ps. In liquids and other condensed systems, the environment might significantly impact the o-Ps lifetime.^2^ In the pick-off annihilation process, the positron annihilates with electrons from neighboring molecules, forming singlet-coupled pairs, such that o-Ps lifetimes can be reduced to a few nanoseconds.^23–25^

The sensitivity of the pick-off lifetimes observed in experiments with the Positron Annihilation Lifetime Spectroscopy (PALS) technique is one of the main motivations to study Ps atoms in materials. Among the experimental techniques related to Positron Annihilation Spectroscopy, Coincidence Doppler Broadening Spectroscopy^26^ (CDBS), Angular Correlation of Annihilation Radiation^23,27^ (ACAR), and Age Momentum Correlation^25,28^ (AMOC) also stand out. In addition to materials sciences, recent interest in positron annihilation is also related to the PET imaging technique and other biomedical applications.^29,30^ The contribution of Ps to annihilation signals is anticipated to support further technological advancements. Notably, the sensitivity of o-Ps lifetimes to molecular oxygen concentrations^31^ offers a promising method for identifying hypoxic regions within tumors,^32^ and the detection of three-photon annihilation events may enable more precise localization of o-Ps annihilation sites in biological tissues.^33,34^ Furthermore, recent experimental advances have demonstrated positronium lifetime imaging in the human brain,^35^ as well as the observation that the degree of entanglement between annihilation photons in matter depends on the annihilation mechanism, with pick-off annihilation producing unentangled photons and direct or parapositronium annihilation yielding maximally entangled photons.^36^ Finally, positron applications to cancer therapy have been considered.^37^

Measurements employing the PALS technique are commonly interpreted with bubble models, which account for o-Ps in molecular materials.^38–40^ According to this model, the o-Ps forms a spherical cavity or “bubble″ within the medium primarily due to Pauli repulsion between the electron of the o-Ps and surrounding electrons. This localized free volume affects the o-Ps lifetime, which is sensitive to the size and distribution of these cavities. The o-Ps annihilation predominantly occurs through pick-off processes, where the positron in o-Ps interacts with an electron from the cavity boundary, resulting in a lifetime that is inversely related to the electron density at the cavity boundary. Consequently, the bubble model correlates o-Ps lifetimes with nanoscale free volumes in materials. Recent refinements to the bubble model, accounting for quantum confinement effects and more realistic electron density distributions, have improved its prediction capability.^20,41^ However, the need to further improve the available models to bridge the gap between predictions and experimental observations was also noted.^9,42^

The description of positrons and Ps atoms in chemically complex environments, such as biological systems, is particularly challenging. We recently proposed a protocol for simulating Ps atoms in water,^43^ combining the sequential Quantum Mechanics/Molecular Mechanics^44^ (s-QM/MM) technique with the Any Particle Molecular Orbital^45,46^ (APMO) method. The latter generalizes electronic structure techniques for systems containing more than one type of quantum species. In our approach, the Ps-water system was treated classically (MM step) employing a force field (FF) based on Coulomb and Lennard-Jones (LJ) potentials to model Ps-solvent and solvent–solvent interactions. The repulsive component of the FF, which emulates the short-range Pauli repulsion, was obtained from the FF for solvated electrons,^47^ while the attractive component was calculated with a Slater–Kirkwood formula.^48^ Once statistically uncorrelated Ps-water configurations were generated in the MM step, APMO calculations were performed in the subsequent QM step, providing estimates for annihilation lifetimes, vertical binding energies, and other properties.

In the present study, we extend our QM/MM model to investigate Ps atoms in methanol and ethanol. Although describing Ps particles in these solvents is an interesting challenge per se, pure alcohols should also be viewed as suitable systems to further develop FFs for Ps, since experimental data for solvated electrons are available.^49–57^ Knowing that QM/MM methods are currently the workhorse of computational biochemistry and biophysics, our ultimate goal would be introducing Ps atoms in the QM/MM framework to describe those quantum particles in complex biological systems. Despite many challenges along this path, the present study can be viewed as an important step.

This paper is organized as follows: In Section 2, we describe the s-QM/MM method, the Ps-solvent force fields adapted for liquids with multiple interaction sites, and provide a summary of the APMO methodology and the enhancement factors. Section 3 begins with a discussion of the solvated electron models developed for methanol and ethanol, which form the basis of the proposed Ps force fields. We then present the classical properties of Ps atoms derived from MC simulations and provide a detailed analysis of the s-QM/MM results, with a special focus on the positronic and electronic densities’ impact on the pick-off lifetimes. Conclusions and perspectives for future work are outlined in Section 4.

## 2 Methods

As outlined above, our QM/MM protocol is based on the s-QM/MM approach, which employs classical Monte Carlo (MC) simulations in the MM step, followed by QM calculations performed for statistically uncorrelated Ps-solvent configurations obtained. In the following, we present the FF used to model Ps-solvent interactions, as well as the APMO methodology used in the QM calculations.

### 2.1 Ps-Solvent Force Field

We perform MC simulations using the Metropolis algorithm implemented in the DICE software,^58^ modeling a system consisting of 500 solvent molecules and a single positronium (Ps) atom in a cubic box with periodic boundary conditions. Methanol and ethanol molecules were treated as rigid bodies, restricting the sampled configuration space to translational and rotational degrees of freedom. We employed the isothermal–isobaric (*NpT*) ensemble at *T* = 298.15 K and *p* = 1 atm, along with standard numerical procedures described in the Supporting Information. Only the trans conformations of methanol and ethanol were considered, with all solvent molecules kept rigid in these conformations throughout the simulations.

Intermolecular interactions were described using a FF composed of Coulomb and LJ potentials. The interaction sites are characterized by effective charges, *q*_*i*_, and the LJ parameters, ϵ_*i*_ and σ_*i*_, where the subscript *i* identifies the solvent and Ps sites. Solvent–solvent interactions were described using the OPLS-UA parametrization,^59^ in which nonpolar hydrogen atoms are integrated into the corresponding carbon interaction sites. As in our previous study for hydrated Ps atoms,^43^ the repulsive part of the LJ potential is obtained from FF for solvated electrons. The solvated electron models also employ LJ parameters ( $\epsilon_{e^{-}}$ and $\sigma_{e^{-}}$ ) and charge $q_{e^{-}}=-1$ to describe the interaction of the negatively charged particle with the solvent. While the electron FF was already available for water, in the present study we need to obtain the LJ parameters for electrons solvated in methanol and ethanol.

Since the OPLS-UA model treats hydrogens in methyl and methylene groups implicitly, dispersion interactions are not assigned to these individual hydrogen atoms. Accordingly, LJ interactions were computed only for Ps-O and Ps-C pairs, while Ps-H interactions were neglected. The Ps-A potential, where A refers to either a carbon or an oxygen atom, is given by

1 $$U_{PsA}\left(r\right)=\frac{C_{12}^{PsA}}{r^{12}}-\frac{C_{6}^{PsA}}{r^{6}}$$

where *r* denotes the interatomic distance. The repulsion and dispersion coefficients are related to the LJ parameters via $C_{6}^{\mathrm{PsA}}=4\epsilon \sigma^{6}$ and $C_{12}^{\mathrm{PsA}}=4\epsilon \sigma^{12}$ respectively, with the standard combination rules $\epsilon =\sqrt{\epsilon_{\mathrm{Ps}}^{A}\epsilon_{A}}$ and $\sigma =\sqrt{\sigma_{\mathrm{Ps}}^{A}\sigma_{A}}$ . In the Lennard-Jones potential, σ is the distance at which the potential changes from repulsive to attractive, while ϵ is the depth of the potential well.

In consistency with our previous study,^43^ and also with phenomenological models for Ps-matter interactions, we assume the repulsive term to be dominated by Pauli repulsion, i.e., the *C*_12_ parameter for Ps is obtained from the solvated electron parameter, $C_{12}^{\mathrm{PsA}}=C_{12}^{e^{-}A}$ . This term plays a central role in the model, as it accounts for the cavitation of the solvent. In turn, the attractive dispersion coefficient was calculated using the Slater–Kirkwood formula^60^

2 $$C_{6}^{PsA}=\frac{2C_{6}^{PsPs}C_{6}^{AA}}{\left(\frac{\alpha_{Ps}}{\alpha_{A}}\right)C_{6}^{AA}+\left(\frac{\alpha_{A}}{\alpha_{Ps}}\right)C_{6}^{PsPs}}$$

where α_Ps_ and α_A_ are the static polarizabilities of Ps and the atom A, respectively, and $C_{6}^{\mathrm{PsPs}}$ and $C_{6}^{\mathrm{AA}}$ are the dispersion coefficients for Ps-Ps and A-A interactions.^48^ Nearly exact values are available for the Ps atom, namely $\alpha_{\mathrm{Ps}}=36a_{0}^{3}$ and $C_{6}^{\mathrm{PsPs}}=207.97\mathrm{Hartree}.a_{0}^{6}$ , while established values for atomic polarizabilities are used for the carbon $\left(\alpha_{C}=11.3a_{0}^{3}\right)$ and oxygen $\left(\alpha_{O}=5.41a_{0}^{3}\right)$ .^61^ Therefore, with both $C_{6}^{\mathrm{PsA}}$ and $C_{12}^{\mathrm{PsA}}$ defined, the LJ parameters for Ps can be determined analytically through the equations

3 $$\sigma_{Ps}^{A}=\frac{1}{\sigma_{A}}{\left(\frac{C_{12}^{PsA}}{C_{6}^{PsA}}\right)}^{1/3},\epsilon_{Ps}^{A}=\frac{1}{\epsilon_{A}}{\left(\frac{C_{6}^{PsA}C_{6}^{PsA}}{4C_{12}^{PsA}}\right)}^{2}$$

In the OPLS-UA parametrization for alcohols, LJ parameters are assigned to all heavy atoms, while for the hydroxyl hydrogen only effective charge is assigned. Specifically, methanol includes the methyl carbon (C­(CH_3_)) and hydroxyl oxygen (O), while ethanol includes the methyl carbon (C­(CH_3_)), methylene carbon (C­(CH_2_)), and hydroxyl oxygen (O). Accordingly, we computed the LJ parameters ( $\epsilon_{\mathrm{Ps}}^{A}$ and $\sigma_{\mathrm{Ps}}^{A}$ ) for each of these heavy atoms and considered their averages to define the FF parameters for Ps. Therefore, the final positronium force field is defined by $\sigma_{\mathrm{Ps}}=\underset{A}{\sum }\sigma_{\mathrm{Ps}}^{A}/N_{\mathrm{LJ}}$ and $\epsilon_{\mathrm{Ps}}=\underset{A}{\sum }\epsilon_{\mathrm{Ps}}^{A}/N_{\mathrm{LJ}}$ , where *N*_LJ_ represents the number of interaction sites in the molecule that have LJ parameters. This procedure was adopted because our goal is to develop a single, universal set of Ps parameters capable of describing its interactions with all atomic sites. Although fully universal parameters will be derived in future work, the use of averaged values over different interaction sites already provides satisfactory results without requiring a more complex parametrization.

### 2.2 Quantum Calculations

The second step of the QM/MM protocol involves multicomponent quantum mechanical calculations, in which the atomic nuclei are treated as fixed point charges within the Born–Oppenheimer approximation, while electrons and positrons are explicitly described as quantum particles. These calculations were performed using the multicomponent APMO method^45,46^ as implemented in the LOWDIN software.^62^ Wave functions were obtained at the APMO Hartree–Fock (APMO/HF) level, and correlation energies (including both electron–electron and electron-positron contributions) were calculated using the generalized second-order APMO propagator method (APMO/P2).^63^

Vertical detachment energies (VDEs) for both the positron and electron were calculated using Koopmans’ theorem (KT), which approximates the VDE as the negative of the energy of the corresponding *p*th singly occupied molecular orbital (SOMO)

4 $${VDE}_{KT}^{\alpha }=-\epsilon_{p}^{\alpha }$$

where $\epsilon_{p}^{\alpha }$ denotes the SOMO energy of the electron (α = *e* ^–^) or positron (α = *e* ^+^). In the framework of multicomponent propagator theory, these KT estimates can be systematically improved by incorporating relaxation and correlation effects via the self-energy correction, $\Sigma_{pp}^{\alpha }\left(\omega_{p}^{\alpha }\right)$ , leading to the equation

5 $${VDE}_{P2}^{\alpha }=-\omega_{p}^{\alpha }=-\epsilon_{p}^{\alpha }-\Sigma_{pp}^{\alpha }\left(\omega_{p}^{\alpha }\right)$$

where $\omega_{p}^{\alpha }$ is the corrected orbital energy obtained by solving the Dyson equation iteratively.

The self-energy is usually expressed as electron–electron (or positron-positron) and electron-positron terms as

6 $$\Sigma_{pp}^{\alpha }\left(\omega_{p}^{\alpha }\right)=\Sigma_{pp}^{\alpha \alpha }\left(\omega_{p}^{\alpha }\right)+\Sigma_{pp}^{\alpha \beta }\left(\omega_{p}^{\alpha }\right)$$

computed at second order as

7 $$\begin{array}{ll} \Sigma_{pp}^{\alpha \alpha }\left(\omega_{p}^{\alpha }\right) & =\underset{j,a\in \alpha }{\sum }\frac{\left|\left\langle pa\right|\right|pj\rangle {|}^{2}}{\omega_{p}^{\alpha }+\epsilon_{a}-\epsilon_{p}-\epsilon_{j}}+\underset{i\neq p,j,a\in \alpha }{\sum }\frac{\left|\left\langle pa\right|\right|ij\rangle {|}^{2}}{\omega_{p}^{\alpha }+\epsilon_{a}-\epsilon_{i}-\epsilon_{j}}+\underset{i,a,b\in \alpha }{\sum }\frac{\left|\left\langle pi\right|\right|ab\rangle {|}^{2}}{\omega_{p}^{\alpha }+\epsilon_{i}-\epsilon_{a}-\epsilon_{b}}\\ & =T_{\mathrm{ORX}}^{\alpha }+T_{\mathrm{PRX}}^{\alpha }+T_{\mathrm{PRM}}^{\alpha } \end{array}$$

and

8 $$\begin{array}{ll} \Sigma_{pp}^{\alpha \beta }\left(\omega^{\alpha }\right) & =\underset{j,a\in \beta }{\sum }\frac{\left|\left\langle pa\right|pj\right\rangle {|}^{2}}{\omega_{p}^{\alpha }+\epsilon_{a}-\epsilon_{p}-\epsilon_{j}}+\underset{\begin{array}{c} i\neq p\\ i\in \alpha \end{array}}{\sum }\underset{j,a\in \beta }{\sum }\frac{\left|\left\langle pa\right|ij\right\rangle {|}^{2}}{\omega_{p}^{\alpha }+\epsilon_{a}-\epsilon_{i}-\epsilon_{j}}+\underset{a\in \alpha }{\sum }\underset{i,b\in \beta }{\sum }\frac{\left|\left\langle pi\right|ab\right\rangle {|}^{2}}{\omega_{p}^{\alpha }+\epsilon_{i}-\epsilon_{a}-\epsilon_{b}}\\ & =T_{\mathrm{ORX}}^{\alpha \beta }+T_{\mathrm{PRX}}^{\alpha \beta }+T_{\mathrm{PRM}}^{\alpha \beta } \end{array}$$

where $T_{ORX}$ terms correspond to orbital relaxation effects and the $T_{PRM}$ and $T_{PRX}$ terms represent pair-removal and pair-relaxation correlation contributions, respectively. The pair-relaxation term $T_{PRX}^{e^{-}‐e^{+}}$ only arises when multiple orbitals are occupied. In the above equations, *a*, *b* and *i*, *j* stand for unoccupied and occupied molecular orbitals indexes running over two-particles Coulomb integral ⟨*ij*|*ab*⟩ and orbital energies ϵ. A detailed derivation of the self-energy correction terms within the APMO framework, as well as illustrative examples of their application to electron-positron systems, can be found in refs. 63,64

Due to the large size of the system represented in our classical MC simulations, comprising a Ps atom and 500 solvent molecules in a cubic box, full quantum mechanical treatment is computationally infeasible for the liquid configurations. To address this, we employed a hybrid QM/MM scheme in which the Ps atom and its nearest solvent molecules were treated at the quantum level during the QM step, while the surrounding environment was represented by a static electrostatic field generated from the same atomic charges used in the classical force field (electrostatic embedding). This embedding scheme has recently been implemented in LOWDIN and has been applied successfully to positronic systems in aqueous environments.^43,65^

### 2.3 Annihilation Rate

The prediction of annihilation rates for positron-electron systems is a stringent challenge, particularly due to the strong correlation between the particle-antiparticle pairs, which demands computationally intensive methods. As a result, it is common practice to incorporate empirical enhancement factors to account for many-body effects.^66–68^ In this work, we extend the application of the QM/MM protocol to solvated Ps atoms in alcohols, with a focus on obtaining pick-off lifetimes using multicomponent APMO wave functions and enhancement factors.

To quantify annihilation, we calculated the spin-averaged two-photon annihilation rate (λ), expressed as^69,70^

9 $$\lambda =\pi r_{0}^{2}c\int \Psi^{*}\left({\mathrm{R⃗}}_{e},{\mathrm{r⃗}}_{p}\right)\delta \left({\mathrm{R⃗}}_{e},{\mathrm{r⃗}}_{p}\right)\Psi \left({\mathrm{R⃗}}_{e},{\mathrm{r⃗}}_{p}\right)d{\mathrm{R⃗}}_{e}d{\mathrm{r⃗}}_{p}$$

where *r*_0_ is the classical electron radius, *c* is the speed of light, and Ψ is the APMO/HF wave function. The operator $\delta \left({\mathrm{R⃗}}_{e},{\mathrm{r⃗}}_{p}\right)=\overset{N}{\underset{i=1}{\sum }}\delta \left({\mathrm{r⃗}}_{i}-{\mathrm{r⃗}}_{p}\right)$ imposes the superposition of the electron and positron coordinates. This allows the annihilation rate to be expressed in terms of orbital density overlaps

10 $$\lambda =\pi r_{0}^{2}c\left(S_{N}+\overset{N_{\alpha }}{\underset{i=1}{\sum }}S_{i}^{\alpha }+\overset{N_{\beta }}{\underset{j=1}{\sum }}S_{j}^{\beta }\right)$$

where *N* = *N*_α_ + *N*_β_ + 1 includes all solvent electrons (*N*_α_ + *N*_β_) plus the unpaired Ps electron (α and β denote spin projections). The term *S*_*N*_ is obtained from the singly occupied electronic orbital, thus describing Ps annihilation. $S_{i}^{\alpha }$ and $S_{j}^{\beta }$ represent overlaps between the positron density and the densities of the doubly occupied electronic orbitals of the solvent molecules, which account for the pick-off process in our model. The core and valence contributions to the latter process can be easily obtained by classifying the electronic orbitals accordingly.

The HF method provides poor estimates for the annihilation rates. To remedy this deficiency without significantly increasing computational cost, we applied corrections involving enhancement factors. In this approach, the integrals appearing in the calculation of the annihilation rates are multiplied by factors, typically denoted as γ, in order to compensate for the underestimation introduced by the Hartree–Fock approximation. In particular, we adopted the parametrization of these factors proposed by Green and Gribakin,^68^ which were fitted to many-body theory (MBT) results for positron annihilation with noble-gas atoms and depend on the energies of the occupied electronic orbitals (ϵ_*i*_),

11 $$\gamma_{i}=1+\sqrt{\frac{1.31}{-\epsilon_{i}}}+{\left(\frac{0.834}{-\epsilon_{i}}\right)}^{2.15}$$

such that the corrected pick-off annihilation rate becomes

12 $$\lambda_{po}=\pi r_{0}^{2}c\left(\overset{N_{\alpha }}{\underset{i=1}{\sum }}\gamma_{i}S_{i}^{\alpha }+\overset{N_{\beta }}{\underset{j=1}{\sum }}\gamma_{j}S_{j}^{\beta }\right)$$

Pick-off lifetimes (τ_po_ = 1/λ_po_) and radial profiles of the pick-off densities were computed using Becke’s multicenter integration algorithm^71^ as implemented in Multiwfn code^72,73^ employing a grid with 250 radial and 5810 angular points.

## 3 Results and Discussion

As outlined in Section 2.1, the FFs for the Ps are constructed using the repulsive coefficients of solvated electron FFs, while the dispersion interactions are calculated via the Slater–Kirkwood formula. Since the FFs for electron-alcohol interactions are not available, we need to develop solvation models for excess electrons in methanol and ethanol. As described below, the FF parameters are obtained from the structure of the first solvation shell, the spin density, radius of gyration, vertical detachment energies, and absorption spectrum.

### 3.1 Solvated Electron

Solvated electrons in polar solvents such as methanol and ethanol are fundamental species of interest in radiation chemistry, photochemistry, and charge-transfer processes. Once generated, typically through radiolysis or photoionization,^55^ the quasi-free electrons become trapped in cavities stabilized by the surrounding solvent molecules.^50,51^ The solvation process, driven by hydrogen bonding networks, dielectric properties, and molecular structure,^54^ strongly influences the spatial distribution of electrons, as well as the binding energy and the spectroscopic properties.^56^ In alcohols, the interplay between hydrophilic hydroxyl groups and hydrophobic alkyl ones creates unique solvation environments that distinguish them from water. Understanding the structure and dynamics of these reactive species is critical for elucidating their reactivity, mobility, and role in ultrafast charge transport phenomena in condensed-phase systems. In this work, we focus on estimating the excess electron cavities and the impact on the Ps FF without attempting a full quantitative treatment of all electronic properties of solvated electrons.

The characteristics of the excess electron cavity are strongly influenced by the LJ parameters, particularly the repulsive parameter $\sigma_{e^{-}}$ , which mainly determines the cavity size, while the Coulomb interaction is maintained through a fixed elementary charge. In this work, we adopted the LJ parameters initially proposed by Ludwig et al.^47^ for the hydrated electron, namely $\epsilon_{e^{-}}=0.08$ kcal/mol and $\sigma_{e^{-}}=4.04Å$ . To investigate the role of cavity size, we also generated alternative FFs for methanol by retaining the same $\epsilon_{e^{-}}$ value and varying $\sigma_{e^{-}}$ across a range of values, 4.0, 4.2, 4.5, 4.7, and 4.9 Å. Based on preliminary studies established for methanol, we restricted our analysis to three of these models, which are referred to as model I $\left(\sigma_{e^{-}}=4.2Å\right)$ , model II $\left(\sigma_{e^{-}}=4.5Å\right)$ , and model III $\left(\sigma_{e^{-}}=4.7Å\right)$ . Structural and electronic properties were explored with the different FFs, which essentially differ in the size of the cavities produced by the repulsive LJ parameters.

In consistency with the s-QM/MM methodology, the molecular mechanics (MM) simulations were performed using classical MC simulations with the DICE code. Simulations were carried out in the NpT ensemble at 298.15 K and 1 atm, using a cubic box containing 500 solvent molecules for both methanol and ethanol. The OPLS-UA force field was employed to model the solvent molecules. From these MC simulations, between 60 and 100 uncorrelated electron-liquid configurations were extracted and subsequently used for the QM calculations. The details of the simulation protocol are available as Supporting Information.

#### 3.1.1 Radial Distribution Functions

The first property analyzed with the different electron solvation models was the solvent structure. The organization of solvent molecules around the excess electron in polar protic solvents such as methanol and ethanol has been elucidated through a range of experimental techniques, including pulse radiolysis,^49^ resonance Raman spectroscopy,^52,74^ electron spin resonance (ESR),^51^ and ultrafast transient absorption spectroscopy.^56^ These studies consistently show that the solvated electron is stabilized within a hydrogen-bonded cavity formed by the inward orientation of hydroxyl (OH) groups from the surrounding solvent molecules. In methanol, resonance Raman and ESR data mentioned above suggest that the cavity accommodates approximately 4–6 solvent molecules, forming a compact, nearly spherical structure with an effective radius of about 2.2–2.4 Å. In ethanol, the first solvation shell appears slightly larger, containing 5–7 molecules and a cavity radius in the range of 2.5–2.7 Å, attributed to the bulkier ethyl side chains. In both solvents, the hydrophobic alkyl groups orient away from the electron center, while the polar OH groups point inward, creating a stabilizing electrostatic and hydrogen-bonding environment. The only difference regarding the orientation of the solvent molecules in the first layer is the direction of the OH group toward the center of the cavity in methanol, while for ethanol, the preferential orientation is that of the molecular dipole. These structural features support a model in which directional hydrogen bonding and solvent geometry cooperatively define the localization and stabilization of the excess electron in alcohols.

The radial distribution functions, *G*(*r*), obtained from our MC simulations are shown in Figure 1. Integration of the electron-center-of-mass radial distribution function, *G*_e‑CM_(*r*), up to its first minimum yields coordination numbers of six methanol molecules for cavity models I and II, while five molecules for model III. These values are consistent with theoretical predictions from ab initio cluster studies and molecular dynamics simulations, which estimate 4–6 molecules in the first solvation shell.^75,76^ Furthermore, the cavity radius determined from the onset of *G*_e‑A_(*r*) was found to be 2.32, 2.45, and 2.44 Å for models I, II, and III, respectively, in fair agreement with the 2.5 Å reported by Mones et al.^75^ These results reinforce that the hydroxyl groups preferentially orient toward the excess electron, as expected. The cavity models for methanol reproduce solvation structures in agreement with experimental observations and with available computational studies. Additional results for $\sigma_{e^{-}}=4.0$ and 4.9 Å are provided as Supporting Information.

**Figure 1:**
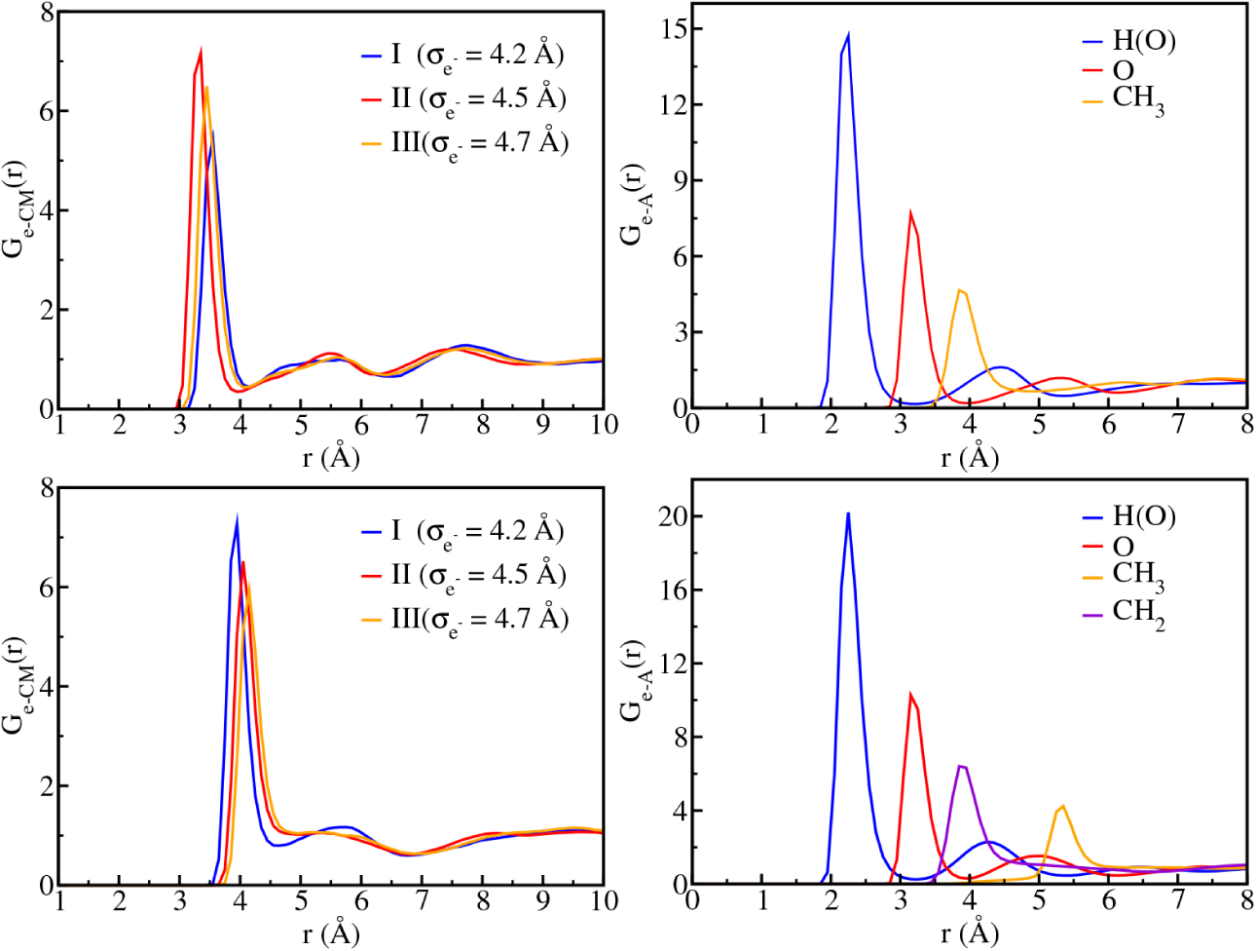
Radial distribution functions *G*_e‑CM_(*r*) between the excess electron and the solvent center of mass, and *G*_e‑A_(*r*) between the electron and selected atomic sites of the solvent molecules: hydroxyl hydrogen (H­(O)), hydroxyl oxygen (O), methylene carbon (C­(CH_2_)), and methyl carbon (C­(CH_3_)). Upper panels show results for methanol, lower panels correspond to ethanol considering the cavity models I $\left(\sigma_{e^{-}}=4.2Å\right)$ , II $\left(\sigma_{e^{-}}=4.5Å\right)$ , and III $\left(\sigma_{e^{-}}=4.7Å\right)$ .

A similar analysis was performed for the excess electron in ethanol. For cavity models I and II ( $\sigma_{e^{-}}=4.2$ and 4.5 Å), the first solvation shell comprised six ethanol molecules, while model III $\left(\sigma_{e^{-}}=4.7Å\right)$ yielded a coordination number of seven. The corresponding cavity radii were determined to be 2.23, 2.72, and 2.83 Å for models I, II, and III, respectively. These results are consistent with available experimental data for the solvated electron in ethanol, which report coordination numbers in the range of 5–7 molecules and cavity radii between 2.5 and 2.7 Å. Overall, the ethanol cavity models also reproduce solvation structures in good agreement with experimental observations.

#### 3.1.2 Spin Density Distributions and Radius of Gyration

The spatial distribution of the excess electron was further analyzed through spin density isosurfaces and radial spin densities. These were computed with QM regions comprising the excess electron and the solvent molecules in the first solvation shell surrounded by 200 MM molecules described by atomic point charges (electrostatic embedding). For methanol, the QM region is composed of 6 molecules in both cavity models, while for ethanol there are 6 and 7 molecules in models I and II, respectively. The calculations were carried out with the DFT/CAM-B3LYP, DFT/M06–2X, and HF methods employing the 6–31++G­(d,p) basis set in all cases using the Gaussian09 package.^77^

Figure 2 displays representative spin density isosurfaces for cavity model II in methanol and ethanol, along with radial spin density distributions obtained for the cavity models I, II, and III. We restrict our discussion to the results obtained with the M06–2X functional, but similar trends were observed for CAM-B3LYP and UHF calculations, as discussed in the Supporting Information. The isosurfaces clearly indicate that the unpaired electron density remains confined within the solvent cavity for both solvents, in consistency with the radial distribution functions. The results also agree with earlier studies, which reported strong localization of the solvated electron in the hydrogen-bond-stabilized cavities.^75,76,78^

**Figure 2:**
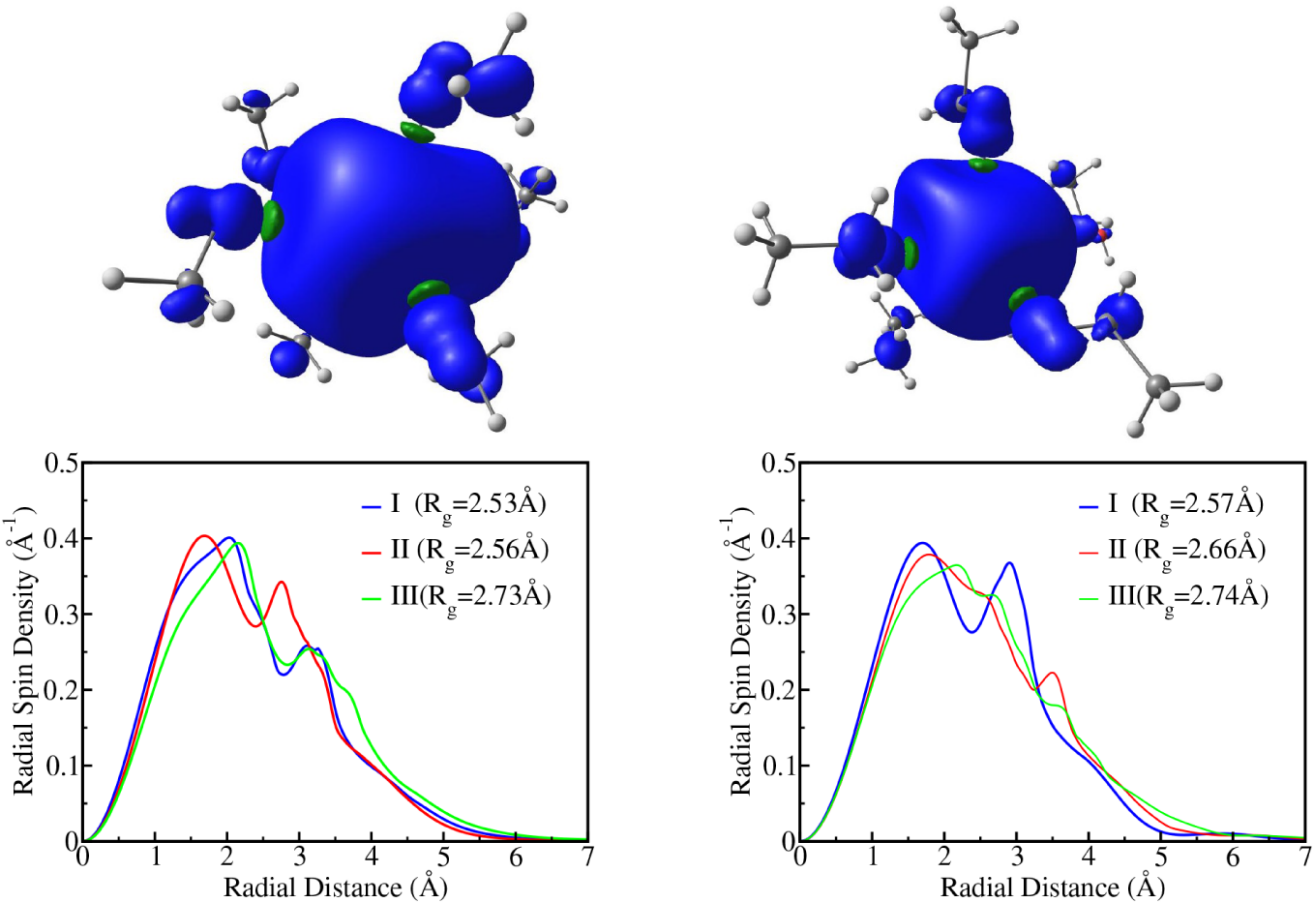
Isosurfaces and radial distributions of spin densities for methanol (left panels) and ethanol (right panels). The radii of gyration (*R*_g_) obtained from the radial distributions are also indicated. Both isosurfaces correspond to model II $\left(\sigma_{e^{-}}=4.5Å\right)$ and were computed using the M06–2X functional. Positive and negative spin density isosurfaces are shown in blue and green, respectively, using the isovalue of 0.0005 au.

The compactness of the spin density varies systematically with the cavity size parameter $\sigma_{e^{-}}$ . For model I, the spin density is more concentrated, indicating a tightly bound, highly localized excess electron. In contrast, model III produces a broader, more delocalized distribution. Model II serves as an intermediate case, although its radial spin density profile and radius of gyration closely resemble those of the cavity model I. The corresponding radius of gyration values for methanol are 2.53, 2.56, and 2.73 Å, for models I, II, and III, respectively, while those for ethanol are 2.57, 2.66, and 2.74 Å. These values were derived directly from the radial spin density distributions and are in reasonable agreement with experimental and theoretical estimates. Tuttle and Golden^79^ reported an experimental value of 2.28 Å, while theoretical studies have yielded a range of values depending on the methodology: Turi’s one-electron pseudopotential models^75^ suggest 2.0 Å, ab initio cluster studies^76^ yield approximately 2.4 Å, and Lan et al.^78^ report a broader range of 2.5–2.8 Å. For ethanol, the same study by Tuttle and Golden reported a radius of gyration of 2.30 Å, indicating that our results are also in reasonable agreement with the experiment, as observed for methanol.

A notable feature of the radial spin density profile for model II is the presence of a shallow local minimum near 2.3 Å, in methanol, which corresponds closely to the peak in the radial distribution function between the excess electron and hydroxyl hydrogen atoms (see Figure 1). This suggests a subtle structural rearrangement at that distance, likely reflecting a balance between localization pressure from hydrogen-bonded OH groups and the electron’s quantum delocalization. In ethanol, a less pronounced decrease in spin density is observed at this same position. This partial reduction in the spin density near the hydroxyl hydrogen is also visible in the isosurfaces, consistent with the features described by Walker et al.^76^ for methanol. In their work, a node in the wave function between the oxygen and the proton leads to a region of decreased spin density near the OH bond, attributed to antibonding character and spin polarization. In our methanol and ethanol isosurfaces, we observe a similar nodal behavior, particularly in methanol, where the effect is more pronounced. For ethanol, this feature persists but is noticeably attenuated, likely due to a broader distribution of the excess electron and the influence of the alkyl chain on the local solvation geometry.

All SOMO orbitals obtained from the QM calculations also exhibit strong localization within the solvent cavity, as shown in the Supporting Information, further confirming the consistency between spin density and orbital-based descriptors of electron confinement. Additional spin density plots and radial distributions obtained using CAM-B3LYP and UHF methods are provided in the SI and reveal trends analogous to those observed with M06–2X, supporting the robustness of the results across electronic structure methods.

#### 3.1.3 Electronic Vertical Detachment Energies

To assess how the cavity structure affects experimentally accessible properties, we analyzed the vertical detachment energies (VDEs) of the excess electron for the different cavity models. Although our approach is not intended to produce quantitatively exact predictions, the relative trends in VDEs provide meaningful insight into the dependence of electron stabilization on cavity size. The QM/MM models comprise 6 or 7 quantum molecules and 200 classical ones, as described in Section 3.1.2.

Recent experimental work using extreme ultraviolet photoelectron spectroscopy with liquid microjets observed VDEs of approximately 3.3 eV for both methanol and ethanol, indicating comparable stabilization of the excess electron in these solvents.^57^ Earlier measurements by Horio et al.^54^ reported slightly lower VDEs around 3.1 eV. In contrast, VDE values obtained for methanol clusters by Kammrath et al.^53^ ranged from 2.0 to 2.5 eV for internally solvated states to 0.2–0.5 eV for surface-bound electrons, highlighting the sensitivity of electron binding energies to solvation environment and system size.

In this study, VDEs were computed using DFT along with the CAM-B3LYP and M06–2X functionals, Koopmans’ theorem (KT), and second-order propagators (P2) with the 6–31++G (d,p) basis set, as summarized in Table 1. In DFT calculations, the VDEs were computed as the total energy difference between neutral and anionic systems, while KT and P2 estimates are based on orbital energies. All VDE values were obtained as ensemble averages over the set of uncorrelated snapshots extracted from equilibrated MC simulations, corresponding to cavity models I, II, and III. Due to the high computational cost of the P2 method, calculations for ethanol were not performed with this method.

**Table 1:** Vertical Detachment Energies (VDEs) in ev of the Excess Electron in Methanol and Ethanol for Cavity Models I $\left(\sigma_{e^{-}}=4.2Å\right)$ , II $\left(\sigma_{e^{-}}=4.5Å\right)$ , and III $\left(\sigma_{e^{-}}=4.7Å\right)$ ^a^

Methanol			
Method	Model I (σ = 4.2 Å)	Model II (σ = 4.5 Å)	Model III (σ = 4.7 Å)
CAM-B3LYP	3.02 ± 0.03	2.90 ± 0.03	2.72 ± 0.03
M06–2X	3.09 ± 0.03	2.91 ± 0.03	2.62 ± 0.03
P2	2.94 ± 0.03	2.82 ± 0.03	2.61 ± 0.03
KT	2.74 ± 0.03	2.62 ± 0.03	2.43 ± 0.03

Ethanol			
Method	Model I (σ = 4.2 Å)	Model II (σ = 4.5 Å)	Model III (σ = 4.7 Å)
CAM-B3LYP	2.76 ± 0.04	2.58 ± 0.03	2.44 ± 0.03
M06–2X	2.87 ± 0.04	2.69 ± 0.03	2.52 ± 0.04

a VDEs Were Computed Using the 6–31++G­(d,p) Basis Set, with a QM Region Comprising the First Solvation Shell and the Remaining Solvent Molecules Represented in the MM Region via OPLS/UA Point Charges.

The observed dependence of vertical detachment energies on the cavity size is consistent with the general understanding of excess electron localization in polar solvents. Smaller cavities (Model I, $\sigma_{e^{-}}=4.2Å$ ) lead to a more compact solvation shell, enhancing electron stabilization through stronger electrostatic and polarization interactions with the surrounding molecules. This effect is clearly reflected in the VDEs computed for both methanol and ethanol, where the smallest cavities yield the highest VDEs, above 3.0 eV in methanol and above 2.7 eV in ethanol, when using CAM-B3LYP and M06–2X functionals. These values closely approach the experimental estimates reported by Horio et al.^54^ and Suzuki et al.^57^ As the cavity size increases (Model III, $\sigma_{e^{-}}=4.7Å$ ), a systematic reduction in VDEs is observed for all electronic structure methods, consistent with a more diffuse electron distribution and reduced solvent stabilization. The VDEs in ethanol are consistently lower than in methanol for similar cavity models, likely due to the bulkier molecular geometry and lower dielectric constant of ethanol, which reduce the overall polarity and compactness of the solvation environment. Among the methods employed, density functional theory generates the highest binding energies, whereas the P2 method yields slightly lower VDEs than DFT. As expected, the KT method underestimates VDEs due to its neglect of correlation, providing lower-bounds.

The computed VDEs span a range of 2.4–3.1 eV, lying between liquid-phase experimental data (≥3.1 eV) and cluster-based results such as those by Kammrath et al.,^53^ which reported lower binding energies (2–2.5 eV). For methanol, we explored the VDE dependence on the size of the QM region (see the SI). As the number of QM molecules was increased from 6 to 14 in the M06–2X calculations, we obtained (3.36 ± 0.03) eV and (3.15 ± 0.04) eV for the cavity models I and II, respectively. We believe these values are reasonably converged because little variation was found for VDEs calculated with 10 and 14 quantum molecules, and the same trends were observed for ethanol (see the SI). Curiously, the model I agrees with the measurement of Suzuki et al.^57^ (3.3 eV) while model II with Horio et al.^54^ (3.1 eV).

#### 3.1.4 Absorption Spectra

To complete the analysis of vertical detachment energies and further characterize the electronic structure of the excess electron, we computed absorption spectra using time-dependent density functional theory (TD-DFT). For each solvent, we obtained the first 20 vertical excitation energies for 60 to 100 uncorrelated liquid configurations. The resulting excitation energies were convoluted using Gaussian functions to reproduce the experimental spectral width and line shape as accurately as possible. We considered the cavity models I and II for both solvents, as well as the CAM-B3LYP and M06–2X functionals along with the 6–31++G (d,p) basis set. The computed absorption spectra obtained with M06–2X for methanol and ethanol are shown in Figure 3, see Supporting Information for CAM-B3LYP results.

**Figure 3:**
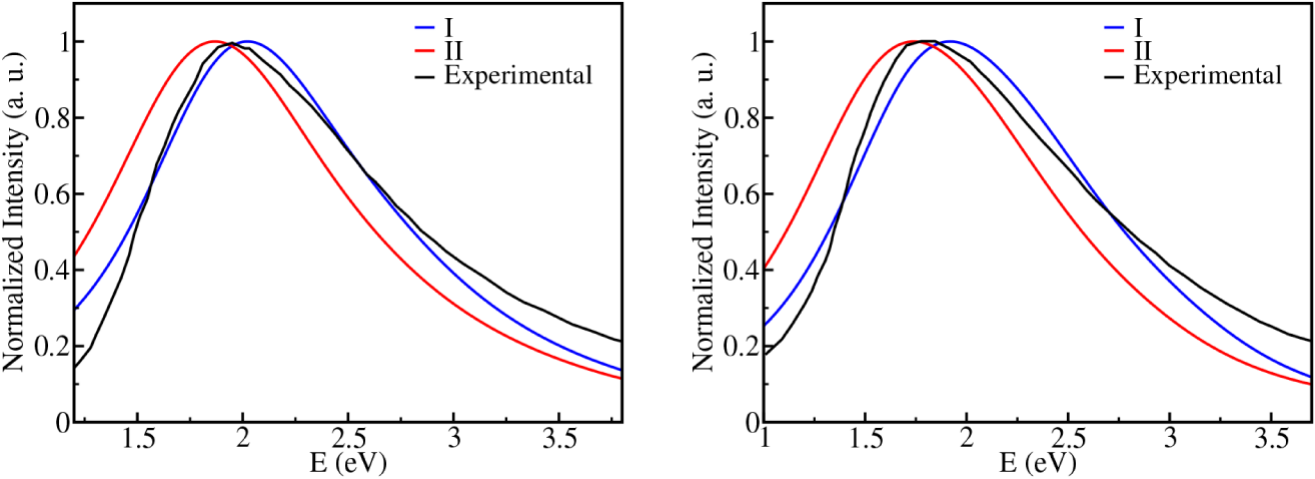
Absorption spectra of the excess electron in methanol (left) and ethanol (right) for cavity models I (blue) and II (red). The spectra were computed using the 6–31++G­(d,p) basis set and M06–2X exchange-correlation functional, with a QM region comprising the first solvation shell and the remaining solvent molecules represented in the MM region via OPLS/UA point charges. Experimental spectra are included for comparison, reproduced from ref. 80 Copyright 1977 American Chemical Society.

In all calculations, the computed spectra have similar shapes, although variations were observed for both the exchange-correlation functional and cavity size. As in previous theoretical studies, the spectra obtained with the range-separated hybrid CAM-B3LYP functional (see Supporting Information) exhibit blue shifts with respect to those computed with M06–2X. These trends persist across the cavity models I and II, indicating that the choice of functional impacts the absorption band even more than the cavity structures considered here. However, the size of the cavity is also relevant: model I $\left(\sigma_{e^{-}}=4.2Å\right)$ , which produces a smaller cavity, yields a blue-shifted absorption maximum due to the stronger spatial confinement of the excess electron. Increasing the cavity size to $\sigma_{e^{-}}=4.5Å$ (model II) facilitates electron delocalization and red shifts the absorption maximum. This behavior is consistent for both methanol and ethanol.

Experimentally, the absorption maximum of the solvated electron has been reported near 1.95 eV for methanol and slightly red-shifted to approximately 1.80 eV for ethanol.^80^ Earlier measurements by Kevan and coworkers using pulse radiolysis established peak positions at 1.95 and 1.88 eV for methanol and ethanol, respectively.^51^ Our simulations accurately reproduce these solvent-specific shifts, absorption maxima obtained with models I and II fall within ± 0.1 eV of the experimental values, depending on the functional employed.

In general, the cavity model I combined with the M06–2X functional provides the best agreement with the experimental VDE obtained by Suzuki et al.^57^ and also with the measured absorption spectra. For methanol, we recalculated the M06–2X spectrum considering 10 and 14 molecules in the QM regions (see Supporting Information). The calculated spectrum was essentially insensitive to the size of the QM region.

### 3.2 Solvated Positronium Atom

The cavity models discussed in Section 3.1 show fair agreement with experimental and theoretical results for the excess electron in methanol and ethanol, specifically with respect to electronic structure, vertical detachment energies, absorption spectra, and solvation shell organization. The cavity models I and II, corresponding to LJ parameters of $\epsilon_{e^{-}}=0.08$ kcal/mol and $\sigma_{e^{-}}=4.2Å$ and 4.5 Å, respectively, showed the closest alignment with the experimental results for the excess electron. These models were therefore adopted as the basis for constructing FFs for the Ps resulting in two models for methanol: I (ϵ_Ps_ = 0.007 kcal/mol, σ_Ps_ = 5.21 Å) and II (ϵ_Ps_ = 0.003 kcal/mol, σ_Ps_ = 5.98 Å). Similarly, for ethanol, we derived the models I (ϵ_Ps_ = 0.007 kcal/mol, σ_Ps_ = 5.20 Å) and II (ϵ_Ps_ = 0.003 kcal/mol, σ_Ps_ = 5.97 Å).

We discuss the results obtained for the classical and quantum simulations of Ps solvated in methanol and ethanol according to our QM/MM protocol. The results for both FF models will be addressed, with special attention to the pick-off lifetimes related to PALS experiments. In accordance with the s-QM/MM methodology, the MM step was performed using classical MC simulations with the DICE code as the first step. These simulations were carried out in the NpT ensemble at 298.15 K and 1 atm, using a cubic box containing 500 solvent molecules for both methanol and ethanol. From these production runs, 70 uncorrelated liquid configurations were extracted and subsequently used for the QM calculations in the second step. The simulation protocol and additional numerical details of the MC simulations are provided in the Supporting Information.

#### 3.2.1 Classical Results

We present in Figure 4 the Ps to center of mass radial distribution function, *G*_Ps‑CM_(*r*), obtained for models I and II. For methanol, the first minimum in *G*_Ps‑CM_(*r*) is located at 6.20 Å and 6.70 Å for models I and II, respectively, defining the extension of the first solvation shell. Similar features are observed for ethanol, where the corresponding minima occur at 6.80 Å and 7.10 Å, indicating slightly larger cavity dimensions with model II in both solvents. The results clearly show that increasing the σ_Ps_ parameter significantly affects the size of the Ps cavity, as expected. Beyond the first minimum, a second solvation shell is discernible and the bulk solvent density (*G*_Ps‑CM_(*r*) ≈ 1) is reached around and beyond 13 Å. The solvation shells comprise approximately 200 solvent molecules, as indicated by the integration of *G*_Ps‑CM_(*r*) up to 13 Å. In turn, integration of *G*_Ps‑CM_(*r*) up to the first minimum gives the coordination numbers of 13 and 16 methanol molecules in the first solvation shell for models I and II, respectively, while 12 and 13 ethanol molecules for the same models. The cavity radius can be estimated from the onset of *G*_Ps‑H(O)_(*r*), yielding values of 2.95 Å and 3.05 Å for methanol, and 2.85 Å and 3.15 Å for ethanol, respectively for models I and II. These onsets of *G*_Ps‑A_ do not show preferential orientation of the sites toward Ps for both solvents. These conclusions are consistent with the hydrophobic nature of Ps observed in the previous study of Ps hydrated resulting in a large cavity.^43^ However, the cavity radii are smaller than the empirical estimates of bubble models, which are around 5 Å for both solvents.^81^

**Figure 4:**
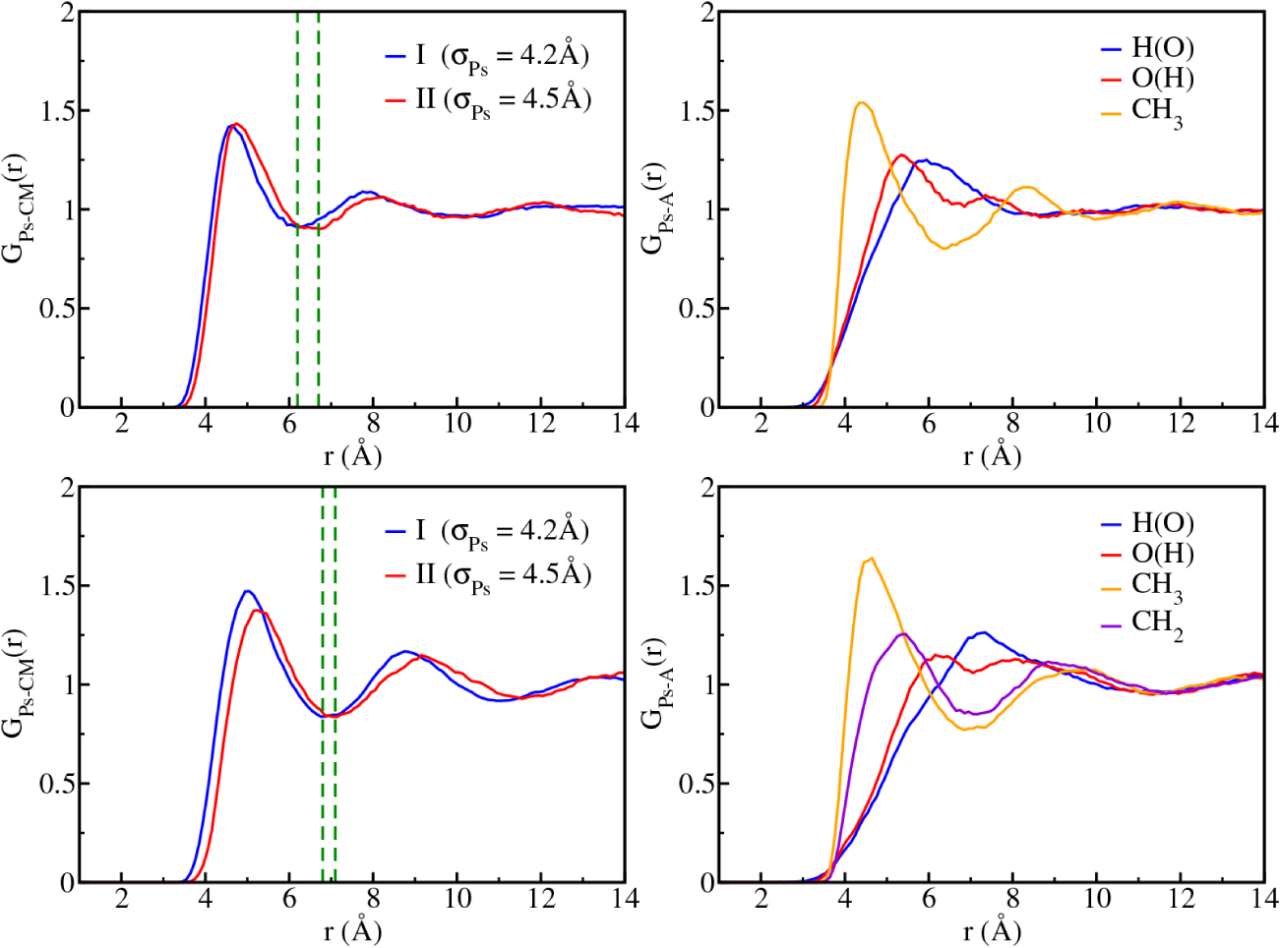
Radial distribution functions for Ps-solvent center of mass pairs (*G*_Ps‑CM_(*r*)) for models I and II, and for Ps-selected atomic site pairs (*G*_Ps‑A_(*r*)) for model II. Upper panels show results for methanol while lower panels correspond to ethanol. Dashed green lines indicate the first solvation shell.

In addition to the solvent structure analysis, we computed the solvation free energy of Ps (Δ*G*) using the Free Energy Perturbation (FEP) method,^82–84^ as in the previous study of hydrated Ps atoms.^43^ The solvation free energy estimates were (2.80 ± 0.02) kcal/mol and (3.23 ± 0.05) kcal/mol for methanol, while (2.41 ± 0.10) kcal/mol and (3.05 ± 0.12) kcal/mol for ethanol, for models I and II, respectively. These positive values indicate that Ps solvation is a nonspontaneous process similar to the Ps solvation in water^43^ for both models. The Δ*G* magnitudes are comparable to those reported for noble gases in water, such as neon (2.48 kcal/mol) and xenon (1.45 kcal/mol), which are prototypical hydrophobic solutes that form clathrate-like structures.^85^

We also estimated the solvation energy (Δ*E*) as the energy difference between the noninteracting and solvated systems

13 $$\Delta E=E_{sol}-E_{free}=\left(E_{Ps-sol}+E_{sol-sol}^{Ps}\right)-\left(E_{sol-sol}^{bulk}\right)$$

In classical simulations, the noninteracting system energy *E*_free_ accounts only for solvent–solvent interactions in the bulk $\left(E_{\mathrm{sol}-\mathrm{sol}}^{\mathrm{bulk}}\right)$ , whereas the energy of the solvated system includes both Ps-solvent interactions (*E*_Ps‑sol_) and the modified solvent–solvent interactions in the presence of Ps $\left(E_{\mathrm{sol}-\mathrm{sol}}^{\mathrm{Ps}}\right)$ . These quantities were averaged over the ensemble of MC configurations and computed for molecules within the simulation cutoff (see ref.^43^ for details). The calculated solvation energies were (−0.80 ± 1.09) kcal/mol and (0.77 ± 1.05) kcal/mol for methanol, while (10.93 ± 1.67) kcal/mol and (10.40 ± 1.58) kcal/mol for ethanol, respectively for models I and II.

The decomposition of these energy terms for model I highlights key differences between the solvents. For methanol, the Ps-solvent interaction is weak (*E*_Ps‑sol_ = −0.10 kcal/mol), and the solvent–solvent interaction energy is essentially unperturbed by the presence of Ps, ( $E_{\mathrm{sol‐sol}}^{\mathrm{Ps}}=-4836.06$ kcal/mol) and ( $E_{\mathrm{sol‐sol}}^{\mathrm{bulk}}=-4836.76$ kcal/mol), resulting in a near-zero total solvation energy. In contrast, ethanol exhibits a similarly weak Ps-solvent interaction, (*E*_Ps‑sol_ = −0.21 kcal/mol), but a notable enhancement in ethanol–ethanol attraction upon solvation, ( $E_{\mathrm{sol‐sol}}^{\mathrm{Ps}}=-4942.07$ kcal/mol) and ( $E_{\mathrm{sol‐sol}}^{\mathrm{bulk}}=-4930.92$ kcal/mol). This reorganization leads to a significantly positive solvation energy in ethanol, despite the overall attractive interaction energy. These contrasting behaviors suggest different solvent structuring around the Ps atom in each medium.

Finally, based on standard thermodynamic relations, the entropy change (Δ*S*) associated with Ps solvation was estimated as

14 $$\Delta S=\frac{1}{T}\left(\Delta E-\Delta G+p\Delta V\right)$$

where *p*Δ*V* is the pressure–volume work associated with cavity formation (see ref.^43^ for details). The resulting entropy changes are (−58.86 ± 15) J/mol·K and (−42.85 ± 15) J/mol·K for methanol, while (111.31 ± 23) J/mol·K and (94.89 ± 22) J/mol·K for ethanol, for models I and II, respectively. The negative entropy changes in methanol reflect the reduction in configurational entropy due to cavity formation and the hydrogen-bonding network. The negligible difference in solvent–solvent interaction energy before and after Ps insertion indicates that the methanol structure resists reorganization, characteristic of hydrophobic solvation and clathrate-like arrangements.^86^

In contrast, the positive entropy changes in ethanol indicate a more disordered solvent environment around the Ps atom. Despite molecular rigidity in the classical MC simulations, the enhanced ethanol–ethanol interactions in the presence of Ps suggest a more favorable reorganization of solvent molecules, likely enabled by the longer alkyl chains and less extensive hydrogen-bonding network compared to methanol. This allows for increased packing efficiency and a greater number of accessible spatial configurations, resulting in a net entropy gain. These findings highlight the critical role of intermolecular solvent restructuring, in determining the thermodynamic signature of Ps solvation in polar solvents.

Although no experimental data are available for these thermodynamic quantities of Ps in the literature, it is possible to compare them with estimated values for the solvation of noble gases in alcohols. These estimations were carried out by us based on the relation between the solvation free energy and the Henry’s law constant, *k*_*H*_. Computer simulations reported in the literature provide *k*_*H*_ values as a function of temperature,^87–89^ allowing a linear fit of Δ*G* versus *T*. From this fit, the slope yields -Δ*S*, while the intercept provides the enthalpy variation Δ*H*, which is approximately equal to Δ*E* (see SI).

The resulting Δ*G* values for He, Ne, Ar, Kr, Xe, and Rn in both methanol and ethanol fall within the range of 2–6 kcal/mol, indicating that the solvation process is non spontaneous. These values are comparable in magnitude to those obtained for positronium. The corresponding Δ*H* ≅ Δ*E* values may be either positive or negative depending on the solute. However, their magnitudes remain below 3 kcal/mol in both solvents, consistent with the computed values for Ps in methanol but smaller than those found for Ps in ethanol. In contrast, all entropy variations are negative, ranging from −80 to −40 J/mol·K, in agreement with the Ps-methanol results but not with the Ps-ethanol case. Altogether, these findings suggest a strong similarity between the solvation behavior of positronium and that of noble gases in methanol, and a more moderate correspondence in ethanol.

#### 3.2.2 Positronic and Electronic SOMO Orbitals

We now focus on the quantum properties of the system as obtained from the second step of our s-QM/MM approach. Because of the composite nature of the Ps-solvent systems, comprising strongly correlated electronic and positronic density components, both the description of correlation effects and the choice of Gaussian basis sets can be challenging. Several basis sets and strategies to choose the expansion centers for the positronic wave function were carefully considered in our previous work on hydrated Ps.^43^ In this work, we employ the procedures that provided a good balance between numerical effort and accuracy.

For the positronic wave function, Gaussian basis functions were placed on the oxygen atoms of solvent molecules within the first solvation shell, using the standard 6–31G++(d,p) basis set for oxygen. This choice reflects the known affinity of positrons for regions of high electronic density arising from lone pairs and can also be supported by condensed Fukui functions.^64^ The electronic wave function was similarly expanded using the 6–31G++(d,p) basis functions centered on the atomic nuclei in the first solvation shell. To better describe the Ps density inside the cavity, additional atomic orbitals are placed at the geometric center of the cavity. Based on the previous work, the aug-cc-pVTZ basis sets for hydrogen was used to expand both the electronic and positronic wave functions at that expansion center. The QM region is composed by the Ps atom and the solvent molecules in the first solvation shell, which are surrounded by an electrostatic embedding containing 200 solvent molecules, up to the bulk density. For methanol, 13 and 16 QM molecules were used with the cavity models I and II, respectively, while 8 QM ethanol molecules were used with both models. This scheme, previously benchmarked for water, was shown to yield reasonably converged results for Ps observables, in particular pick-off lifetimes and VDEs.

The positronic and electronic singly occupied molecular orbitals (SOMOs) were analyzed for an ensemble of uncorrelated liquid configurations. Representative examples of SOMOs are shown in Figure 5 for methanol and ethanol. The positronic and electronic SOMO densities can be understood as the Ps density in our model because the doubly occupied molecular orbitals describe the neutral solvent. Interestingly, our results indicate Ps densities located either inside the cavity or at the boundary of the QM and MM regions, that is, at the surface of the cluster corresponding to the first solvation shell. Although such surface states were absent in our previous simulations of Ps in water, they were frequently found in the present study. We understand that surface states arise from the artificial boundary between the QM and MM regions. The lack of Pauli repulsion effects tends to overly delocalize the electronic density (electron spill-out error^90^ and hence the Ps density. While we did not further explore the formation of surface Ps states, they were considered artifacts of the QM/MM model and disregarded. Our model was built to describe Ps particles in thermodynamic equilibrium with the solvent, so only cavity states were taken into consideration.

**Figure 5:**
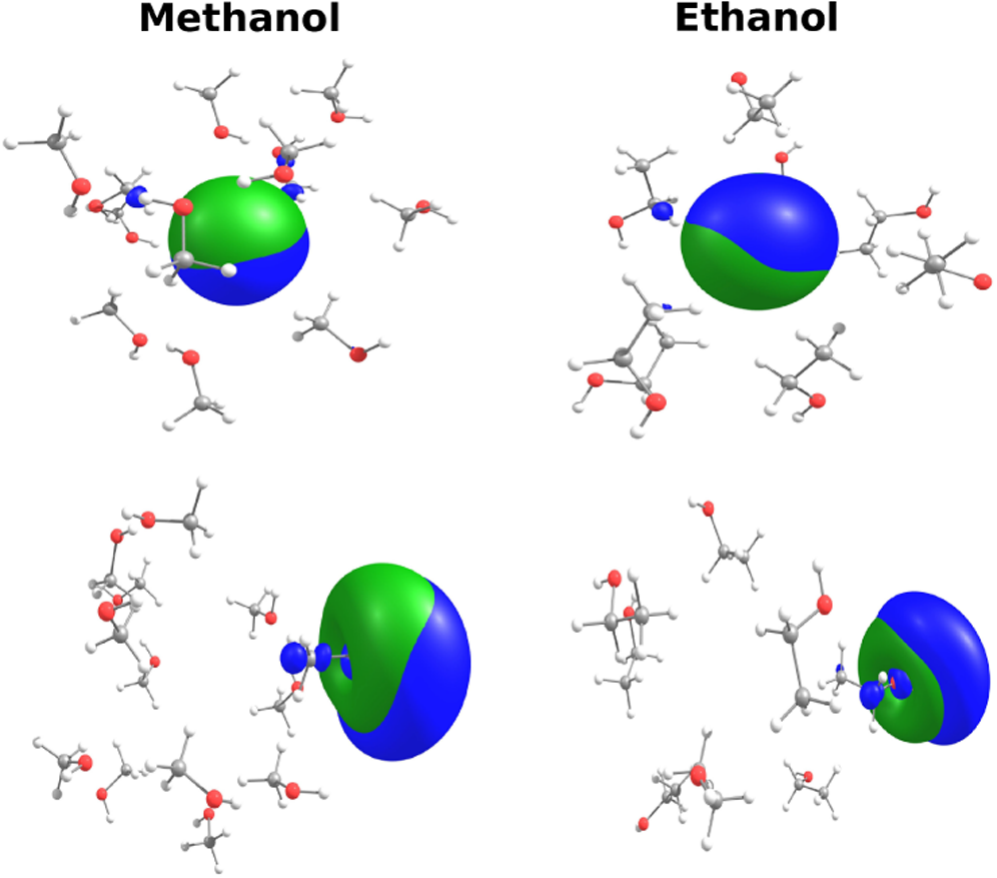
Isosurfaces of electronic (blue) and positronic (green) SOMO orbitals of Ps in methanol (left panels) and in ethanol (right panels) for representative uncorrelated liquid configurations of cavity (top panels) and surface (upper panels) states. The isosurfaces were represented with the isovalue 0.03.

The radial probability densities (RPDs) available in the Supporting Information offer additional insight into the nature of the SOMO orbitals illustrated in Figure 5. In configurations corresponding to cavity states, both the positronic and electronic components exhibit highly overlapping RPDs, which should be expected for Ps atoms. In contrast, surface states are characterized by separated positronic and electronic RPD maxima, reflecting a weaker spatial overlap and thus a less strongly bound Ps complex. Only for the physically meaningful cavity states, we computed RPDs averaged over the ensemble of uncorrelated Ps-solvent configurations corresponding to cavity states. The results are summarized in Table 2. The positions of the RPD maxima (*r*_max_) are essentially the same for electronic and positronic SOMOs in both solvents. Small differences are observed between models I and II for both solvents. Model II yields slightly larger *r*_max_ values, by no more than 0.05 Å, consistent with the larger cavity sizes obtained with this FF. These trends further confirm the sensitivity of Ps localization to subtle changes in cavity structure and can shed some light on the interpretation of Ps properties.

**Table 2:** Ensemble Averages for Electronic (*e* ^–^) and Positronic (*e* ^+^) Radial Probability Density Maximum (RP*D*_max_) and Maximum Position (*r*_max_) for the Models I and II of Both Solvents^a^

Solvent	Model	${\mathrm{RPD}}_{\max}^{e^{+}}\left({Å}^{-1}\right)$	$r_{\max}^{e^{+}}\left(Å\right)$	${\mathrm{RPD}}_{\max}^{e^{-}}\left({Å}^{-1}\right)$	$r_{\max}^{e^{-}}\left(Å\right)$
Methanol	I	0.482 ± 0.002	1.598 ± 0.008	0.484 ± 0.002	1.626 ± 0.006
	II	0.480 ± 0.002	1.610 ± 0.006	0.478 ± 0.002	1.636 ± 0.005
Ethanol	I	0.915 ± 0.002	1.587 ± 0.008	0.491 ± 0.004	1.618 ± 0.006
	II	0.467 ± 0.002	1.641 ± 0.008	0.476 ± 0.002	1.665 ± 0.006

a We computed RPDs averaged over the ensemble of uncorrelated Ps-solvent configurations corresponding to cavity states.

#### 3.2.3 Vertical Detachment Energies

In our model, the VDE corresponds to the energy required to remove either the positron or the electron from the Ps-solvent complex in a vertical process, that is, without nuclear relaxation of the solvent configuration. Considering the computational cost, VDEs were computed with Koopmans’ theorem for the electronic and positronic SOMOs obtained from APMO/HF calculations. The electronic and positronic basis sets, as well as the QM/MM models employed in the calculations, are described in Section 3.2.2. The results shown in Table 3 were also averaged over the set of uncorrelated configurations for both methanol and ethanol, corresponding to cavity states.

**Table 3:** Electronic (*e* ^–^) and Positronic (*e* ^+^) VDEs Estimated with Koopmans’ Theorem for the Models I and II of Both Solvents^a^

Solvent	Model	${\mathrm{VDE}}^{e^{-}}\left(\mathrm{eV}\right)$	${\mathrm{VDE}}^{e^{+}}\left(\mathrm{eV}\right)$
Methanol	I	4.66 ± 0.05	4.77 ± 0.05
	II	4.75 ± 0.04	4.68 ± 0.04
Ethanol	I	4.76 ± 0.04	4.69 ± 0.04
	II	4.60 ± 0.05	4.75 ± 0.05

a We computed VDEs averaged over the ensemble of uncorrelated Ps-solvent configurations corresponding to cavity states.

For both solvents, the modest differences between the cavity models I and II (around 1.0 eV) suggest that Ps binding is fairly insensitive to small variations of the FF parameters. When comparing solvents, we observe no systematic preference for methanol or ethanol in terms of stabilizing the Ps complex. The VDEs fluctuate within the same narrow range (4.60–4.77 eV), and the observed variations mainly reflect thermal and structural fluctuations in the ensemble. Importantly, the similarity between electron and positron detachment energies further emphasizes the strong spatial overlap of the SOMO orbitals, as previously discussed in the analysis of the radial probability densities. Interestingly, the VDEs obtained in the alcohol solvents are consistently higher than those previously reported for Ps solvated in water.^43^ This increase in the VDEs can be attributed, at least in part, to the high overlap between the electronic and positronic SOMO densities observed in the spatial distributions of these orbitals.

Overall, the results confirm that both cavity models provide a reliable description of Ps solvation in methanol and ethanol, with a mild impact on the calculated VDEs. Given the small magnitude of the differences and their proximity to the statistical error, we conclude that models I and II are fairly equivalent in terms of VDEs.

#### 3.2.4 Pick-Off Lifetime

We now turn attention to the pick-off lifetime (τ_*po*_ = 1/λ_*po*_), which is the main physical property of interest. The pick-off annihilation rate, λ_*po*_, was computed using the doubly occupied electron orbitals according to eq 12. For clarity, we discuss the corresponding inverse quantity, 1/λ_*po*_, which we refer to as the pick-off lifetime. This quantity is sensitive to the overlap between the positron density and the electron density of surrounding solvent molecules, and it is central to PALS measurements. The electronic and positronic basis sets, as well as the QM/MM models employed in the calculations, are described in Section 3.2.2. In view of the computational cost, we only considered APMO/HF calculations.

Table 4 presents the pick-off lifetimes computed at the APMO/HF level $\left(\tau_{\mathrm{po}}^{\mathrm{HF}}\right)$ without any empirical correction, and also APMO/HF corrected with the previously reported enhancement factors $\left(\tau_{\mathrm{po}}^{\mathrm{ef}}\right)$ to account for electron-positron correlation effects.^68^ For comparison, several sets of experimental data are available in the literature. Reported pickoff lifetimes for methanol include 3.58 ns,^23^ 3.00 ns,^25^ 3.94 ns,^91^ 3.50 ns,^92^ 3.18 ns,^93^ and 3.33 ns.^94^ For ethanol, the corresponding experimental values are 3.50 ns,^23^ 3.59 ns^25,92^ and 3.81 ns.^91^

**Table 4:** Pick-Off Annihilation Lifetimes (in ns) Calculated with the APMO/HF $\left(\tau_{\mathrm{po}}^{\mathrm{HF}}\right)$ Method and Employing Enhancement Factors $\left(\tau_{\mathrm{po}}^{\mathrm{ef}}\right)$ for Models I and II of Both Solvents^a^

Solvent	Model	τ_*po*_(ns)	$\tau_{po}^{ef}\left(\mathrm{ns}\right)$
Methanol	I	19.75 ± 0.97	4.32 ± 0.21
	II	21.47 ± 0.77	4.72 ± 0.17
Ethanol	I	15.97 ± 0.57	3.57 ± 0.13
	II	21.46 ± 0.93	4.78 ± 0.22

a We computed pick off annihilation lifetimes averaged over the ensemble of uncorrelated Ps-solvent configurations corresponding to cavity states.

The uncorrected Hartree–Fock results significantly overestimate the pick-off lifetimes, as expected, predicting values between 16 and 22 ns. These discrepancies are consistent with the well-known limitations of mean-field treatments of electron-positron interactions, which tend to underestimate the contact density responsible for annihilation. However, when the enhancement factors are applied, the computed lifetimes drop to values in much better agreement with experimental results for both solvents and both cavity models. The comparison between the calculated lifetimes and the experimental data is somewhat difficult for methanol due to the discrepancy among the reported pick-off lifetimes. For this solvent, the largest deviation is approximately 24%, whereas for ethanol the discrepancy is less than 10%, and the reasons behind the much larger disagreement observed for methanol remain unclear to us. In any case, the results obtained with cavity model I consistently show better agreement with experiment for both solvents. Our corrected pick-off value for methanol (4.32 ns) shows the best agreement with the 3.94 ns reported by Gray et al.^91^ (9.6%) and the worst agreement with the 3.00 ns measured by Castellaz et al.^25^ (44%). For ethanol, our result (3.57 ns) shows a worst agreement of 6.3% and a best agreement of less than 1%.

Another relevant point is the difference between the results obtained with models I and II for both methanol and ethanol. Model I yields shorter pick-off lifetimes because the overlap between the positron density and the electronic density of the solvent molecules is favored by the more compact cavity. Even modest differences in cavity size, of the order of 0.1Å, translate into significant variations in τ_*po*_. For methanol, an increase in cavity radius of 3.3%, from model I to model II, increases the corrected pick-off lifetimes by 9.2%, while for ethanol the variation of 10% in radius increases the lifetime by 34%. This behavior is consistent with the RPDs of the positronic densities, shown as red solid lines in SI, which have maxima around 1.6Å for both solvents (see also Table 2). For both solvent and models, the cavity radii are approximately 3Å, a region where the positron densities decrease fast, making the overlap with the solvent electronic density sensitive to the cavity radius.

Taking all the QM/MM results into account, it is clear that the cavity model I performs better than the model II, since it is consistently in similar or better agreement with the experimental data for the electronic VDE, electronic absorption spectra, and the pick-off lifetimes for both solvents. Nevertheless, for any given model, the annihilation lifetimes can still be sensitive to the choice of basis set, as discussed in our previous work on water.^43^ We carried out additional calculations for methanol with different sets of atomic orbitals at the center of the cavity, while keeping the 6–31++G­(d,p) basis set on the atomic centers of the solvent molecules. Our best estimate of the corrected pick-off lifetime, (3.58 ± 0.20) ns, was obtained by placing hydrogenic 6–31++G­(d,p) sets inside the cavity to expand the electronic and positronic wave functions. Although this basis set is not necessarily superior to estimate electronic properties, it gives rise to a slightly higher positron density around *r* ≈ 3Å, compared to the aug-cc-pVTZ basis set, thus increasing the overlap with the solvent electronic density.

If we consider the average value of the measured Ps lifetimes in methanol (3.42 ns), it is evident that the pick-off lifetime obtained with the hydrogenic 6–31++G­(d,p) basis set inside the cavity significantly improves the agreement with the experimental data (error smaller than 5%). However, conclusions should be made with special attention. The annihilation model relies on many independent variables, namely the FF parameters that carve the Ps cavity, the choice of enhancement factors and even the choice basis sets to expand the positronic and electronic wave functions. Since the annihilation lifetimes are experimental information for the Ps-solvent system, one could suitably play around with the model variables to reproduce the experimental pick-off lifetimes for any particular solvent. However, this is not our main goal. The pure liquids should be viewed as starting points to build FFs which, at least in principle, would be transferable to heterogeneous complex environments. Our protocol, even with the hydrogenic aug-cc-pVTZ basis set inside the cavity, agrees with the pick-off measurements for water, methanol, ethanol, and acetonitrile (to be published) within 20% (apart from the two lowest experimental results for methanol, as discussed above). In a sense, this level of agreement is remarkable because we used enhancement factors obtained for monoelectronic atoms^68^ without any further optimization. Once we have parametrized FFs to describe systems containing hydrogen, carbon, oxygen and nitrogen atoms, we are in a position to explore several other pure solvents and even simple heterogeneous systems, taking the challenge to further optimize and survey the transferability of our Ps interaction model. The present results for methanol and ethanol are interesting per se, as they were obtained with modern simulation techniques, but they should also be regarded as an important step toward our larger goal.

## 4 Conclusions and Perspectives

In this work, we have extended a QM/MM protocol to describe the structure, energetics, and annihilation characteristics of Ps atoms in polar protic solvents, specifically methanol and ethanol. This effort builds upon our previous work on Ps in water and represents an important step toward the transferable and systematic modeling of Ps in increasingly complex condensed-phase environments.

Our classical MC simulations with newly developed force fields for Ps-solvent interactions revealed well-defined cavity structures whose size and organization depend sensitively on the LJ parameters used. These structural features were directly reflected in the computed solvation thermodynamics, supporting the picture of Ps as a hydrophobic probe that induces nanoscale cavities in solution. The cavity sizes obtained here were in close agreement with previous estimates for hydrated Ps, and consistently smaller than those predicted by empirical bubble models.

Multicomponent QM calculations based on the APMO/HF method revealed two characteristic Ps localization patterns, cavity and surface states, with the latter identified as spurious artifacts of QM region truncation. The analysis of SOMO isosurfaces and RPDs demonstrated a strong spatial overlap between the positron and electron in cavity states, validating the physical consistency of our approach. Ensemble-averaged VDEs were found to be remarkably robust with respect to cavity size, reflecting the compact nature of the Ps wave function and its insensitivity to modest variations in solvent structure. In contrast, the pick-off annihilation lifetimes turned out to be sensitive to cavity size, with model I yielding shorter lifetimes and better agreement with experimental PALS data for both methanol and ethanol. The application of enhancement factors is essential to achieve reasonable agreement with experiment, confirming their effectiveness when combined with multicomponent quantum mechanical techniques.

Altogether, this study supports the transferability of our QM/MM protocol to predict key observables associated with Ps behavior in solution. By combining realistic solvation structures with physically grounded quantum modeling, we offer a practical and extensible framework for Ps simulations across different chemical environments. Looking forward, this methodology opens the door to several exciting directions. The extension of Ps modeling to aprotic, amphiphilic, or biologically relevant solvents will provide new opportunities to probe nanoscale solvation phenomena. Moreover, the calculation of experimentally accessible observables beyond lifetimes, such as CDBS or ACAR signatures, will further bridge the gap between theory and experiment. Ultimately, the integration of positronium into QM/MM frameworks widely used in biophysics and materials science could pave the way for a deeper understanding of the annihilation of Ps in complex systems.

## Supplementary Material



## Notes

Additional details about the classical and quantum simulations performed for the electron and Ps solvated (PDF)
