# Supplementary material for: Positronium Atoms Solvated in Liquid Alcohols: A Multicomponent Quantum Mechanics/Molecular Mechanics Approach
Source: J Phys Chem B. 2025 Nov 20;129(48):12503–17. doi: 10.1021/acs.jpcb.5c06150 (PMC12683637; doi:10.1021/acs.jpcb.5c06150)
Supplement: Supplementary file 1 [file jp5c06150_si_001.pdf]

# Supporting Information
# "Positronium Atoms Solvated in Liquid Alcohols: a Multicomponent Quantum Mechanics/Molecular Mechanics Approach"

Leonardo Martins[1], Mateus Bergami[2], Jorge Charry[3], Andres Reyes[4], Kaline Coutinho[1] and Márcio T. do N. Varella*[1]

[1]Instituto de Física, Universidade de São Paulo, Rua do Matão 1371, CEP 05508-090 São Paulo, SP, Brazil
[2]Department of Engineering and Physics, Karlstad University, SE-65188 Karlstad, Sweden
[3]Department of Physics and Materials Science, University of Luxembourg, L-1511 Luxembourg City, Luxembourg
[4]Department of Chemistry, Universidad Nacional de Colombia, Av. Cra. 30 #45-03, 111321 Bogotá, Colombia

## 1 Classical simulations

The Monte Carlo (MC) simulations were performed in the $NpT$ ensemble ($T = 298.15$ K and $p = 1$ atm) for systems composed of 500 solvent molecules and a single solute particle, representing either a positronium (Ps) atom or an excess electron. All molecules were treated as rigid bodies throughout the simulations, with only the trans conformations of methanol and ethanol being considered. Solvent–solvent and solute–solvent interactions were modeled using Lennard-Jones (LJ) and Coulomb potentials, with OPLS-UA parameters adopted for the alcohol molecules. The LJ parameters for the Ps atom and for the solvated electron were obtained as described in the main text.

Periodic boundary conditions and the minimum image convention were applied to avoid edge effects in the simulation box. A spherical cut-off with a radius of 12-15 Å (corresponding to 50% of the box length) was used to evaluate explicit pairwise interactions. The sum of interactions due to all molecules outside the sphere is replaced by a long-range correction, based on a continuum model.

Each simulation started with a thermalization phase of $2.5 \times 10^7$ MC steps, followed by production runs consisting of $1.0 \times 10^8$ and $3.0 \times 10^8$ MC steps. All MC simulations were performed using the Metropolis sampling algorithm as implemented in the DICE software. The systems were equilibrated over $2.5 \times 10^7$ MC steps, followed by production runs consisting of $1.0 \times 10^8$ and $5.0 \times 10^8$ MC steps for Ps and electron solvated MC simulations. The average densities obtained during the simulations ranged from $0.76 \pm 0.01$g/cm$^3$ to $0.77 \pm 0.01$g/cm$^3$ for methanol and remained around $0.76 \pm 0.01$g/cm$^3$ for ethanol. These results demonstrate excellent agreement with the experimental densities of the respective pure liquids, validating the simulation protocol for both Ps and electron solvated systems.

*mvarella@if.usp.br

## 2 Spin density distributions and radius of gyration

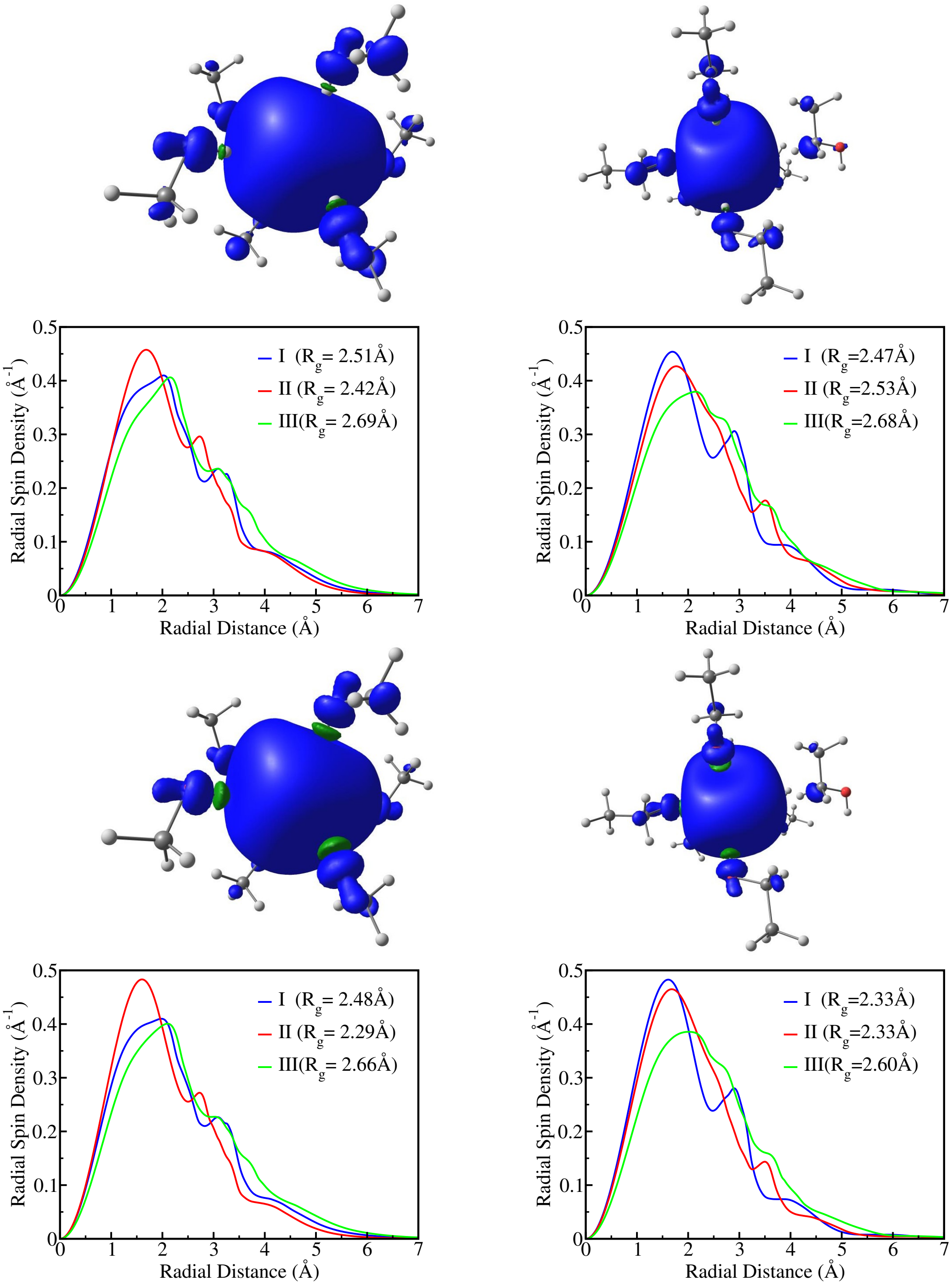


Figure S1: Isosurfaces and radial distributions of spin densities for methanol (left panels) and ethanol (right panels). The radii of gyration ($R_g$) obtained from the spin density radial distributions are also indicated. Both isosurfaces correspond to model II ($\sigma_{e^-} = 4.5$ Å) and were computed using the CAM-B3LYP (top panels) and HF (upper panels). Positive and negative spin density isosurfaces are shown in blue and green, respectively, using the isovalue of 0.0005 a.u.

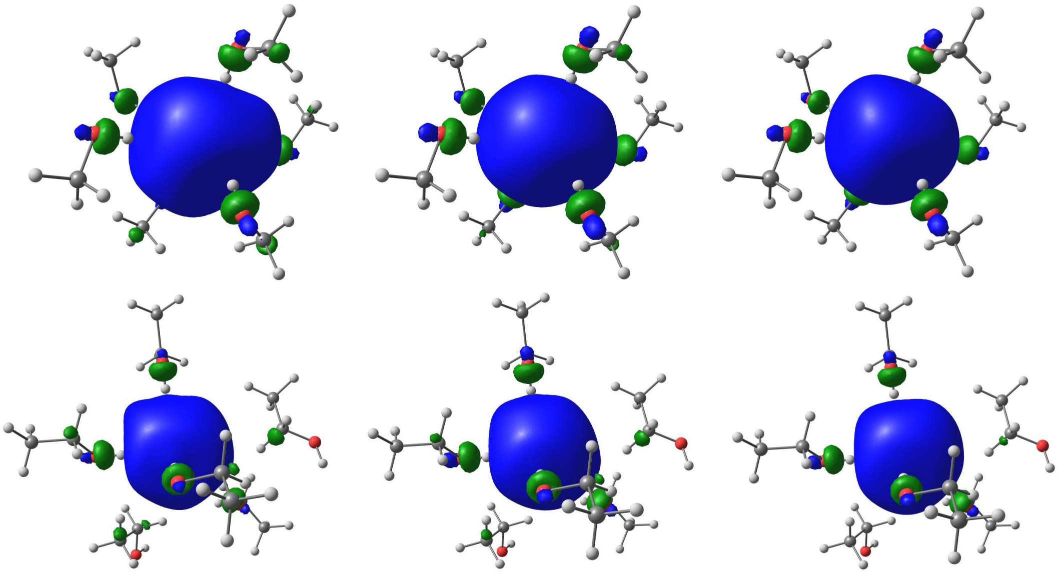

Figure S2: Isosurfaces of SOMO orbitals for the solvated electron in methanol (top panels) and ethanol (upper panels) for cavity model II. These orbitals were computed using M06-2X (left panels), CAM-B3LYP (center panels), and HF (right panels) with the basis set 6-31++G(d,p). Positive and negative isosurfaces are shown in blue and green, respectively, using the isovalue of 0.03 a.u.

# 3 Vertical dettachment energies

| **Methanol** | | |
|---|---|---|
| QM region | Model I ($\sigma = 4.2$ Å) | Model II ($\sigma = 4.5$ Å) |
| first shell | $3.09 \pm 0.03$ | $2.91 \pm 0.03$ |
| 10 MeOH | $3.28 \pm 0.03$ | $3.10 \pm 0.03$ |
| 14 MeOH | $3.36 \pm 0.03$ | $3.15 \pm 0.04$ |
| **Ethanol** | | |
| QM region | Model I ($\sigma = 4.2$ Å) | Model II ($\sigma = 4.5$ Å) |
| first shell | $2.87 \pm 0.04$ | $2.69 \pm 0.03$ |
| 10 EtOH | $3.04 \pm 0.04$ | $2.81 \pm 0.04$ |
| 14 EtOH | $3.10 \pm 0.04$ | $2.89 \pm 0.04$ |

Table S1: Vertical detachment energies (VDEs) of the excess electron in methanol (MeOH) and ethanol (EtOH) for cavity models I ($\sigma_{e^-} = 4.2$ Å) and II ($\sigma_{e^-} = 4.5$ Å). VDEs were computed using the 6-31++G(d,p) basis set and M06-2X exchange-correlation functional, with a QM region comprising 10 and 14 molecules and the remaining solvent molecules represented in the MM region via OPLS/UA point charges.

# 4 Absorption Spectra

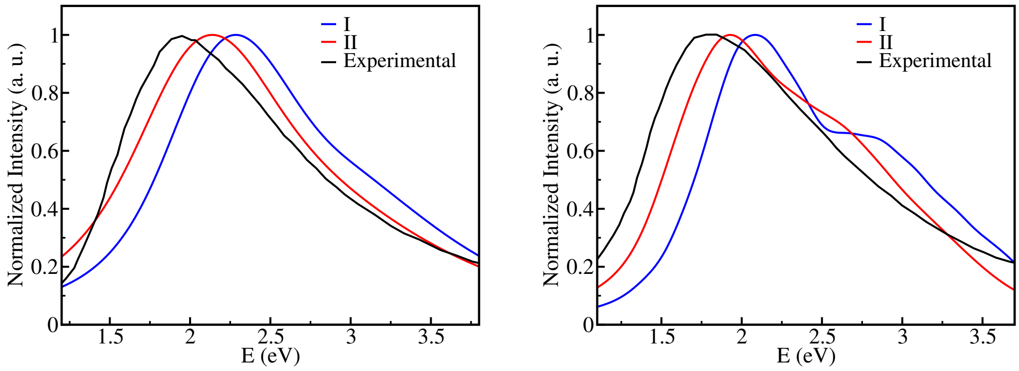


Figure S3: Absorption spectra of the excess electron in methanol (left panel) and ethanol (right panel) for cavity models I (blue) and II (red). The spectra were computed using the 6-31++G(d,p) basis set and CAM-B3LYP exchange–correlation functional, with a QM region comprising the first solvation shell and the remaining solvent molecules represented in the MM region via OPLS/UA point charges. Experimental spectra are included for comparison, reproduced from Ref. 80. Copyright 1977 American Chemical Society.

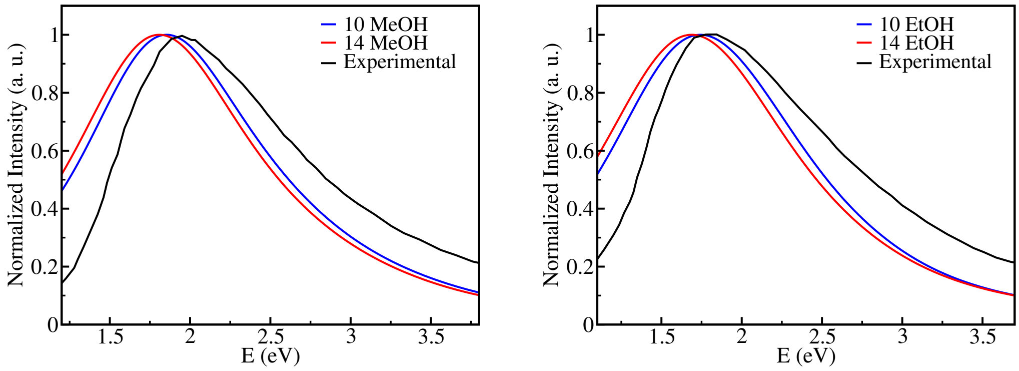


Figure S4: Absorption spectra of the excess electron in methanol (left panel, MeOH) and ethanol (right panel, EtOH) for cavity model II considering 10 (blue) and 14 (red) solvent molecules in the QM region. The remaining solvent molecules are represented in the MM region via OPLS/UA point charges. These spectra were computed using the 6-31++G(d,p) basis set and M06-2X exchange–correlation functional. Experimental spectra are included for comparison, reproduced from Ref. 80. Copyright 1977 American Chemical Society.

## 5 Solvation of Noble Gases in Alcohols

| Methanol | | | |
|---|---|---|---|
| Atom | $\Delta G$ (kcal/mol) | $\Delta E$ (kcal/mol) | $\Delta S$ (J/mol·K) |
| He | $5.814 \pm 0.010$ | $1.978 \pm 0.069$ | $-53.840 \pm 0.925$ |
| Ne | $5.636 \pm 0.015$ | $1.730 \pm 0.089$ | $-54.816 \pm 1.090$ |
| Ar | $4.558 \pm 0.018$ | $0.061 \pm 0.101$ | $-63.102 \pm 1.206$ |
| Kr | $4.081 \pm 0.019$ | $-0.666 \pm 0.106$ | $-66.617 \pm 1.261$ |
| Xe | $3.356 \pm 0.019$ | $-1.666 \pm 0.095$ | $-70.471 \pm 1.097$ |
| Rn | $2.540 \pm 0.019$ | $-2.861 \pm 0.106$ | $-75.792 \pm 1.236$ |
| **Ethanol** | | | |
| Atom | $\Delta G$ (kcal/mol) | $\Delta E$ (kcal/mol) | $\Delta S$ (J/mol·K) |
| He | $5.685 \pm 0.020$ | $2.311 \pm 0.109$ | $-47.346 \pm 1.286$ |
| Ne | $5.499 \pm 0.020$ | $1.985 \pm 0.117$ | $-49.306 \pm 1.414$ |
| Ar | $4.426 \pm 0.023$ | $0.353 \pm 0.124$ | $-57.145 \pm 1.457$ |
| Kr | $3.947 \pm 0.024$ | $-0.341 \pm 0.129$ | $-60.175 \pm 1.514$ |
| Xe | $3.210 \pm 0.026$ | $-1.381 \pm 0.136$ | $-64.423 \pm 1.570$ |
| Rn | $2.370 \pm 0.025$ | $-2.605 \pm 0.126$ | $-69.808 \pm 1.440$ |

Table S2: Computed thermodynamic properties for the solvation of noble gases in methanol and ethanol, derived from the temperature-dependent Henry's law constants reported in Refs. [87-89].

## 6 Positronic and Electronic SOMO Orbitals

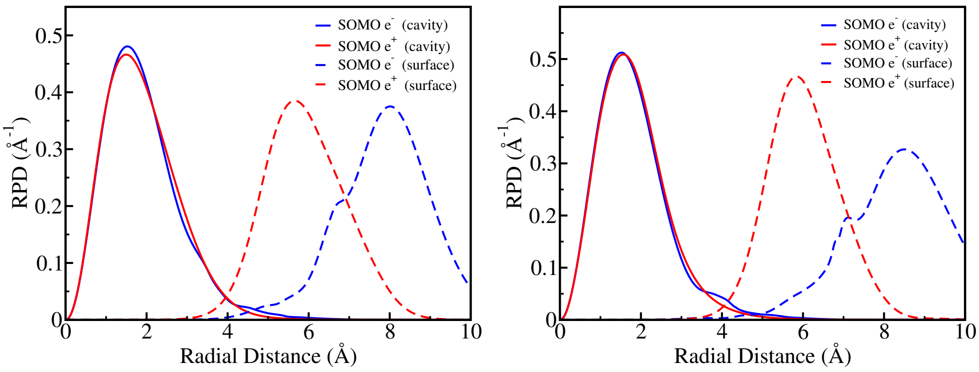


Figure S5: Radial probability density (RPD) of electronic ($e^-$, blue) and positronic ($e^+$, red) SOMO orbitals of Ps in methanol (left panel) and in ethanol (right panel) for the representative uncorrelated liquid configurations of cavity (solid lines) and surface (dashed lines) states represented in Fig. 5.
